# Ten-year survival of pancreas cancer with liver metastases treated by intraoperative and long-term intraperitoneal gemcitabine. A case report

**DOI:** 10.1016/j.ijscr.2023.108313

**Published:** 2023-05-09

**Authors:** Paul H. Sugarbaker, Mark A. Steves

**Affiliations:** a Program in Peritoneal Surface Malignancy, Washington Cancer Institute, Washington, DC, USA; b Department of Surgery, MedStar Washington Hospital Center, Washington, DC, USA

**Keywords:** Peritoneal metastases, Pancreatico-duodenectomy, Intraperitoneal chemotherapy, NIPEC, HIPEC, Case report

## Abstract

**Introduction and importance:**

Progress in the management of pancreas cancer has been slow to occur. Resection of the primary cancer in the head of the pancreas is possible and has become a standard of care in operable patients. Unfortunately, long-term survival after this extensive surgical procedure is nearly nonexistent.

**Case presentation:**

A 55-year-old man was diagnosed with cancer within the pancreas head. He underwent a successful pancreaticoduodenectomy. Hyperthermic intraoperative intraperitoneal chemotherapy (HIPEC) with gemcitabine was added in an attempt to eliminate cancer cells present within the peritoneal space at the time of the resection. Also, six cycles of normothermic intraperitoneal chemotherapy (NIPEC) delivered through an intraperitoneal port were completed. The patient developed a solitary liver metastasis which was removed with adequate margins. The patient remains alive and well and working ten years following treatments.

**Clinical discussion:**

Pancreas cancer shows treatment failures on peritoneal surfaces, as liver metastases, and as systemic and distant lymph nodal disease. The pharmacology of intraperitoneal gemcitabine suggests that it can eliminate peritoneal metastases as a site for treatment failure. Radical surgery can remove lymph nodes in and around the malignancy that are likely to cause a recurrence. Eliminating other sites of treatment failure in this patient allowed the liver resection to result in a long-term survival.

**Conclusions:**

In patients with resectable cancer of the head of the pancreas, local-regional and distant peritoneal recurrence may be reduced as a result of HIPEC and NIPEC gemcitabine being added to the treatments. Additional chemotherapy agents are available to supplement the intraoperative and long-term intraperitoneal gemcitabine treatments. A strategy for bidirectional (both intravenous and intraperitoneal) chemotherapy for pancreas cancer remains as a viable option for improved survival.

## Highlights

• Despite radical surgery pancreas adenocarcinoma has a poor prognosis.
• Local recurrence with or without peritoneal metastases occurs in 60 % of patients.
• Perioperative hyperthermic intraperitoneal gemcitabine can eliminate stray cancer cells.
• Combined intraperitoneal and intravenous chemotherapy may improve outcome.

## 1 Materials and methods

Data on this patient was prospectively recorded and then retrospectively reviewed at an academic institution. This research work has been reported in line with the SCARE 2020 criteria [1]. This study was registered as a case report on the www.researchregistry.com website with UIN 8902. Written permission was obtained from the patient to write this case report.

## 2 Case presentation

January 2013. At age 55, this otherwise healthy male noted a 20-pound weight loss and jaundice. A CT showed a resectable mass in the head of the pancreas, clear CT of the lungs and no additional abdominal or pelvic abnormalities. The superior mesenteric vein was immediately adjacent the pancreatic mass but was not narrowed. The superior mesenteric artery was not involved. Ultrasound-guided biopsy of the mass at the time of esophago-gastro-duodenoscopy showed cells compatible with pancreatic cancer.

The patient signed an informed consent for pancreaticoduodenectomy, hyperthermic intraoperative intraperitoneal chemotherapy (HIPEC) gemcitabine, insertion of an intraperitoneal port and six cycles of normothermic intraperitoneal (NIPEC) gemcitabine administered through the intraperitoneal port.

February 8, 2013. Through an upper abdominal midline incision, a pancreatico-duodenectomy was performed. The abdominal exploration was negative for liver, peritoneal and lymph nodal metastases. The resection was uneventful. HIPEC gemcitabine was performed intraoperatively at 42 °C for 60 min. An open HIPEC method was used [2]. The dose of gemcitabine was 2100 mg (1000 mg/m^2^) diluted in 3.15 L (1.5 L/m^2^) of 1.5 % dextrose peritoneal dialysis solution. The HIPEC gemcitabine was well tolerated [3].

**Fig. 1:**
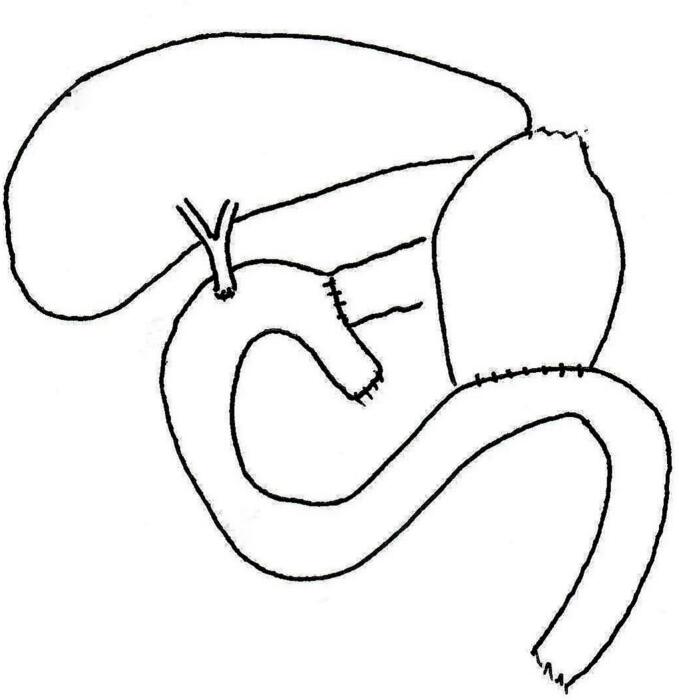
Reconstruction of the upper gastrointestinal tract following pancreaticoduodenectomy. A choledochojejunostomy, pancreaticojejunostomy and gastrojejunostomy were used.

Following HIPEC gemcitabine, the gastrointestinal tract was reconstructed with a pancreatico-jejunostomy, a choledocho-jejunostomy and a gastro-jejunostomy (Fig. 1). Greater omentectomy was performed in order to minimize the possibility of intraperitoneal catheter occlusion. Upon completion of the gastrointestinal reconstruction, the abdomen was lavaged with three liters of an antibiotic solution. The skin of the abdomen was prepped with povidone iodine solution. After these efforts to remove possible intraoperative bacterial contamination, an intraperitoneal port was placed. The tip of the tubing from the intraperitoneal port was placed in the pelvis [4].

Three closed-suction drains were placed in the right subhepatic space, beneath the pancreatico-jejunostomy and in the pelvis. The abdomen was closed in a routine manner. The patient's postoperative course was uneventful. He was discharged to home on his eleventh postoperative day. Pathology report showed a 3 cm greatest diameter mass with a positive margin on the superior mesenteric vein. None of the 5 lymph nodes were positive for cancer.

After one month recuperation, the patient returned to the oncology nursing unit to begin six cycles of NIPEC gemcitabine. The intraperitoneal gemcitabine dose was 2100 mg (1000 mg/m^2^) in 1 L of 1.5 % dextrose peritoneal dialysis solution. The intraperitoneal gemcitabine was given on days one, eight and 15 of a 30-day cycle. All six cycles were administered.

The patient was followed in the Cancer Institute Clinic on a six-monthly basis with chest, abdomen and pelvic CT.

November 2018. The CT performed 5 years and 9 months after pancreatico-duodenectomy showed a 2.5 cm in greatest diameter filling defect in segment four of the liver. The CT showed no pulmonary lesions and the other abdominal and pelvic structures to be normal. A PET-CT showed the segment four liver lesion but did not suggest other sites of disease. Upper and lower gastrointestinal endoscopy were unremarkable.

November 13, 2018. The patient underwent an open wedge excision of the segment four liver lesion. By cryostat section and on permanent histopathologic study, the resected specimen was compatible with metastatic pancreas cancer. Ultrasound of the liver showed no additional pathology. A limited surgical exploration of the abdomen and pelvis was unremarkable. The patient recovered without incident and was discharged on his fifth postoperative day. The patient was placed back into follow-up.

December 6, 2022. Patient underwent an uneventful incisional hernia repair. A surgical site infection required 2 months for complete resolution.

March 2023. At 10 years of follow-up following the pancreaticoduodenectomy with HIPEC and NIPEC gemcitabine and four years after the liver resection, the patient remains well with no evidence of disease. He reports that he is still working.

### 2.1 Pharmacologic study of intraperitoneal gemcitabine

**Fig. 2:**
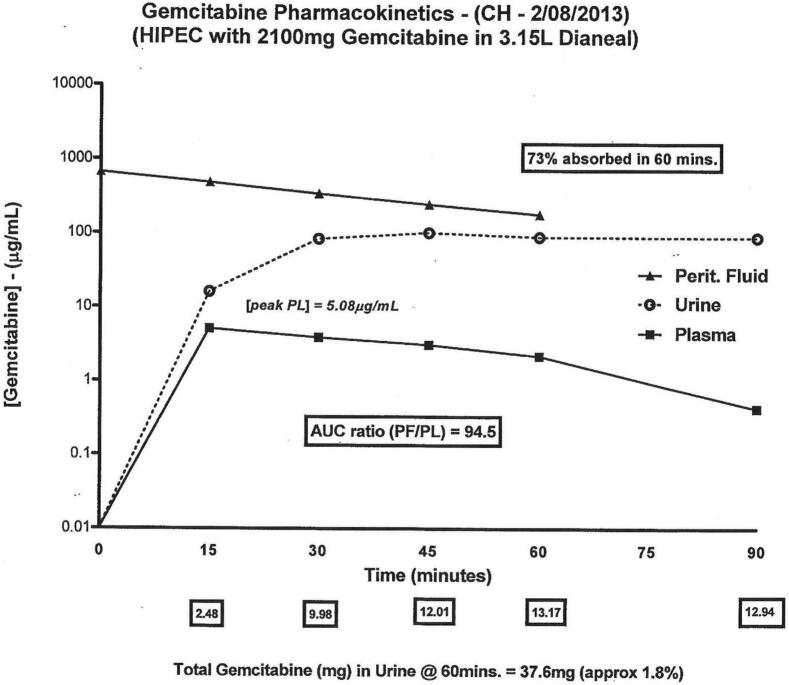
Pharmacokinetic study of hyperthermic intraoperative intraperitoneal (HIPEC) gemcitabine in a 55-year-old man following pancreaticoduodenectomy.

While the HIPEC gemcitabine was performed by the open method samples of blood, chemotherapy solution from within the peritoneal space and urine were taken at 15-minute intervals for 60 min. Blood and urine samples were also available at 90 min. The concentrations of gemcitabine were determined by high pressure liquid chromatography (HPLC). Fig. 2 shows the concentration over time of these samples. The concentration times time for intraperitoneal gemcitabine was 94.5 times higher than the plasma concentration. The peak plasma level of gemcitabine was 5.08 μg/mL. At 60 min, 73 % of the intraperitoneal gemcitabine had penetrated the abdominal and pelvic surfaces and entered the body compartment.

## 3 Discussion

**Fig. 3:**
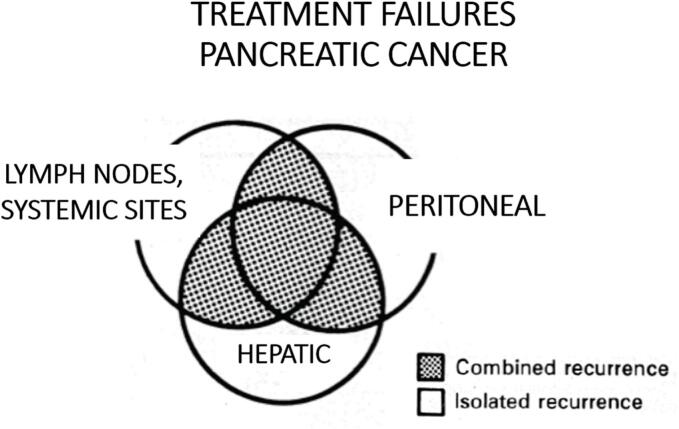
Conceptualization of treatment failures in patients with pancreas cancer. A percentage of patients have isolated recurrence as peritoneal carcinomatosis, hepatic metastases, or lymph nodal disease, but most patients have combined recurrence. Successful adjuvant modalities must be designed to treat all sites of treatment failure. A combination of intraperitoneal and intravenous (bidirectional) chemotherapy is recommended.

The natural history of resectable pancreas is much the same as other abdominal-pelvic gastrointestinal cancers. One notable difference is the exceptionally poor prognosis of resected pancreas cancer. A long-term survivor is extremely unusual [5]. Winter et al. reported only a few percent of the 1423 patients treated at The Johns Hopkins Hospital to show 10-year survival. The disease has an initial pattern of metastatic spread by three different pathways as illustrated in Fig. 3. Dissemination can occur by one or all these pathways of dissemination. Liver metastases may be limited, as in the patient presented, or multiple to all segments of the liver. Likewise, lymphatic dissemination to retroperitoneal nodes may be absent, limited or extensive. Dissemination into the peritoneal spaces with local recurrence progressing around the head of the pancreas resection site is documented in approximately 60 % of patients [6]. The heated gemcitabine administered in the operating room is designed to mitigate against local-regional and peritoneal surface dissemination. The cancer cells lost from the specimen, from dissection very close to the primary tumor and from the surgical trauma to the primary cancer specimen may be destroyed by HIPEC gemcitabine.

Much of the intraperitoneal gemcitabine instilled into the peritoneal space as HIPEC or NIPEC gemcitabine leaves the peritoneal space via the portal blood and may help destroy micrometastases that would, in the absence of intraperitoneal gemcitabine, progress within the liver parenchyma. Similarly, intraperitoneal gemcitabine moving from the peritoneal space to the body compartment will be taken up into the lymphatic system and reduce the likelihood of retroperitoneal nodal progression.

### 3.1 Augmentation of local-regional gemcitabine cytotoxicity by heat

Although the liver metastases and lymphatic metastases can be targeted by effective systemic chemotherapy, the locally disseminated cancer cells will be entrapped in scar tissue and accessed little or not at all by systemic chemotherapy. In contrast, the HIPEC gemcitabine is directed directly at free pancreas cancer cells and cancer cells loosely adherent to raw tissues within and surrounding the resection site. This intraperitoneal chemotherapy should be maximally effective for spilled tumors for two reasons. First, as shown in Fig. 2, the gemcitabine is locally dose intensive with a high concentration of drug within the peritoneal space. Second, the gemcitabine is administered with 42 °C heat. The heat alone (42 °C for 60 min) is by itself cytotoxic to cancer cells [7]. Also, the heat will augment the cytotoxicity of the gemcitabine [8].

### 3.2 Intraperitoneal gemcitabine is expected to have systemic cytotoxicity

Sabbatini et al. has suggested that the systemic concentration of intraperitoneal gemcitabine may be too low to cause cell kill of cancer cells at systemic sites [9]. We see from Fig. 2 that the intraperitoneal gemcitabine is locally dose intensive at the peritoneal surface but also the systemic concentration reaches a peak plasma level of 5.08 μg/mL. These systemic levels of gemcitabine should be active. However, protocols that follow these initial efforts with intraperitoneal chemotherapy should supplement the systemic effects of gemcitabine chemotherapy with systemic chemotherapy such as 5-fluorouracil and cisplatin. Bidirectional (intravenous and intraperitoneal) chemotherapy is theoretically superior considering the pattern of failure of pancreas cancer.

Finally, the use of HIPEC gemcitabine and NIPEC gemcitabine has been shown to be safe. This patient supports this information regarding safety [3]. The intraoperative and long-term intraperitoneal drug did not interfere with normal intestinal function. Also, the intraperitoneal port has caused few complications. Fastidious antibacterial prophylaxis with abdominal antibiotic irrigation and cleansing of the skin with povidone iodine are an essential preparation to port placement. Adhesions surrounding the intraperitoneal catheter are rarely seen because of the frequent administration of chemotherapy through the port. Also, the majority of the abdomen is not involved in an adhesive process for the dissection and surgical trauma is limited to the structures in and around the pancreas.

One interpretation of this favorable long-term survival is a partial but not totally complete treatment of the three routes of dissemination of pancreas cancer. Lymph node metastases did not occur and seeding the resection site with pancreas cancer cells did not occur. Perhaps some but not all of the cancer cells that entered the portal venous blood were destroyed. One focus of disease within the portal blood and liver parenchyma did escape eradication by gemcitabine treatment and required surgical removal.

## Consent

Written informed consent was obtained from the patient for publication of this case report. A copy of the written consent is available for review by the Editor-in-Chief of this journal on request.

## Provenance and peer review

Not commissioned, externally peer-reviewed.

## Ethical approval

Review Board has determined that a case report of less than three [3] patients does not meet the DHHS definition of research (45 CFR 46.102(d)(pre-2018)/45 CFR46.102(l) (1/19/2017)) or the FDA definition of clinical investigation (21 CFR 46.102(c)) and therefore are not subject to IRB review requirements and do not require IRB approval.

## Funding

Data management and secretarial support provided by Foundation for Applied Research in Gastrointestinal Oncology.

## Guarantor

Paul H. Sugarbaker.

## Research registration number

This study was registered as a case report on the www.researchreg istry.com website with UIN 8902.

## CRediT authorship contribution statement

Paul H. Sugarbaker and Mark A. Steves: study concept or design, data collection, data analysis or interpretation, writing the paper, final approval.

## Conflicts of interest

Paul H. Sugarbaker and Mark A. Steves have no conflicts of interest to declare.
